# Examination of Shape Variation of the Skull in British Shorthair, Scottish Fold, and Van Cats

**DOI:** 10.3390/ani13040614

**Published:** 2023-02-09

**Authors:** Ozan Gündemir, Tomasz Szara, Ebru Eravci Yalin, Murat Karabagli, Zihni Mutlu, Osman Yilmaz, Serkan Kemal Büyükünal, Milos Blagojevic, Pere M. Parés-Casanova

**Affiliations:** 1 Department of Anatomy, Faculty of Veterinary Medicine, Istanbul University-Cerrahpasa, 34500 Istanbul, Turkey; 2 Department of Morphological Sciences, Institute of Veterinary Medicine, Warsaw University of Life Sciences-SGGW, 02-776 Warsaw, Poland; 3 Department of Surgery, Faculty of Veterinary Medicine, Istanbul University-Cerrahpasa, 34500 Istanbul, Turkey; 4 Department of Anatomy, Faculty of Veterinary Medicine, Van Yüzüncü Yıl University, 08783 Van, Turkey; 5 Department of Food Hygiene and Technology, Faculty of Veterinary Medicine, Istanbul University-Cerrahpasa, 34500 Istanbul, Turkey; 6 Department of Anatomy, Faculty of Veterinary Medicine, University of Belgrade, 11000 Belgrade, Serbia; 7 Escola Agrària del Pirineu, Finca Les Colomines (Bellestar), 25711 Montferrer i Castellbò, Catalonia, Spain

**Keywords:** geometric morphometrics, shape analysis, veterinary anatomy, taxonomy

## Abstract

**Simple Summary:**

From the taxonomic point of view, it is important to reveal the interspecific and interracial differences in the shape of the skull. This study revealed differences in the shape of the skulls of three different cat breeds. The differences generally occurred around the orbit. It has been shown that the shape of the orbit’s edge is a distinctive feature that differentiates the skulls of cats.

**Abstract:**

A variety of skull shapes are frequently used for discrimination between animal species, breeds, and sexes. In this study, skulls of three different breeds of cats were examined by the geometric morphometric method, with the aim of revealing skull shape differences. For this purpose, 27 cats (6 British Shorthair, 7 Scottish Fold, and 14 Van cats) were used. The skulls of cats were modeled by computed tomography. Geometric morphometrics was applied using dorsal (8 landmarks, 63 semilandmarks) and lateral (8 landmarks, 63 semilandmarks) skull projections on these models. Centroid size differences between the breeds were statistically insignificant. However, the differences in shape were statistically significant for both the dorsal view and lateral view. Shape variation was less in the British Shorthair than in other breeds. Shape differences generally occurred around the orbit. In the skull of Scottish Folds, the orbit was situated more caudally than in other breeds. The British Shorthair had the largest orbital ring. In dorsal view, the Scottish Fold had the largest orbital diameter. The orbital ring of Van cats was smallest in both dorsal and lateral views. In the canonical variate analysis, it was seen that the breeds were separated from each other. The shape difference in the skull between different cat breeds could be revealed by geometric morphometrics. The results of this study provide useful information for taxonomy.

## 1. Introduction

The skull protects the encephalon, the cranial parts of the respiratory and digestive systems, and some sensory organs. Differences between species and sexes are more marked in the skull than in the other parts of the skeleton [1]. Hence, it is commonly used in taxonomic studies [2]. The shape of the skull of various species of mammals is a frequent subject of scientific research [3,4]. In cats and dogs, the zygomatic process (*processus zygomaticus)* of the frontal bone (*os frontale)* does not reach the zygomatic arch (*arcus zygomaticus)*. Its role is taken over by the orbital ligament (*ligamentum orbitale*). Cats also have very large orbits and a strong mandible [5].

Van cats are an endemic breed belonging to the Van district in Turkey. They have unique features, namely heterochromatic eyes and a completely white coat [6]. British Shorthairs have a stocky bodies and round faces. Their eyes are round and large. British Shorthairs are very similar to the Scottish Fold cat. However, the British Shorthair’s ears are more erect, and the facial structure is longer in profile [7]. The Scottish Fold is a purebred cat originating from Australia. Their most distinctive feature is their forward-folding ears [8,9].

In recent years, besides traditional morphological and morphometric studies, geometric morphometrics has been carried out to reveal the anatomical differences between animal species, breeds, and sexes [10,11]. While traditional morphometry reveals the difference in linear measurements, geometric morphometrics analyzes the shape of structures [12,13]. With this method, both two-dimensional and three-dimensional samples can be examined. Geometric morphometrics is used in many disciplines, especially in anatomy and anthropology [14].

The shape difference between the skull of the wolf and the German Shepherd was investigated by the geometric morphometric method [3]. This study found that the differences in shape between the two species were most often expressed in the parietal, occipital, zygomatic, temporal bones, and the ramus of the mandible. The authors reported that the skulls of the wolf and the German Shepherd differed significantly in shape. In another study, the shapes of dingo skulls from different regions of Australia were examined [15]. Demircioglu et al. [16] analyzed shape differences between ram and sheep skulls. They also detected a significant sexual dimorphism of the skull. Furthermore, other authors proved dimorphic features of canine skulls [17]. However, the literature lacks studies comparing the shape of the skull between cat breeds. In this study, the shape variations of the skull of British Shorthair, Scottish Fold, and Van cats were investigated.

## 2. Materials and Methods

### 2.1. Animals

In the study, computed tomography (CT) scans of 27 cat skulls (6 British Shorthair, 7 Scottish Fold, and 14 Van cats) were used. The age of cats was between 2 and 7 years (Table 1). The examined animals were clinically healthy. Cases with skull anomalies or with incomplete bone development were rejected. Samples were obtained from Van Yüzüncü Yıl University, Van Cat Research and Application Center, and Istanbul University-Cerrahpasa, Faculty of Veterinary Medicine, Animal Hospital.

#### D Modeling

Computed tomography scans of the head were taken using Siemens Somatom Scope vc30b and Siemens Somatom Sensation 16 systems. Scanning parameters for all samples were as follows: slice thickness 0.6 mm, 110 kV, and 28 mA, and total scanning time was approximately 14 s. The resulting images were saved in DICOM format and transferred to the workstation. The 3D rendering of the bones was performed using Syngo CT VB20 software (Siemens Healthcare, Erlangen, Germany).

### 2.2. Geometric Morphometric Analysis

**Figure 1:**
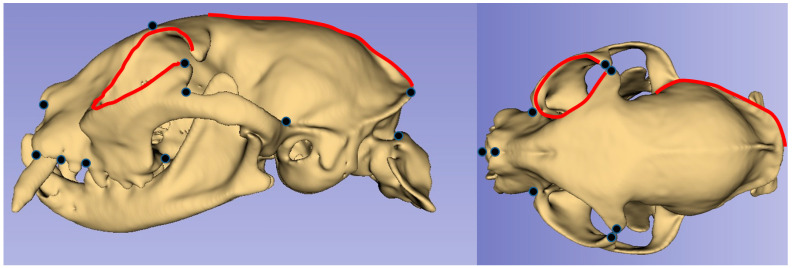
Landmarks and semilandmarks.

The images were converted to the “tps” format using tpsUtil (version 1.74) software [18]. A total of 8 landmarks and 63 semi-landmarks for dorsal view and 11 landmarks and 41 semi-landmarks for lateral view were used (Figure 1). Semilandmarks were used for the border of the orbit and along the borders of the temporal fossa (the external sagittal, nuchal, and temporal crests). Here, TpsDig2 (version 2.32) was used for landmark operations [19].

### 2.3. Statistical Analysis

MorphoJ ver. 1.07 software was used for the statistical part of the geometric morphometric analysis [12]. The landmark file was imported into MorphoJ, and “Procrustes fit” was applied first. Then, the samples were divided into groups (British Shorthair, Scottish Fold, and Van cats). A generalized procrustes analysis was applied to the imported landmark data before analysis. Principal component analysis (PCA) was performed to determine the shape variations among cat skulls. Shape and centroid size amongst breeds were compared with procrustes ANOVA. Canonical variates analysis (CVA) was used to reveal the differences between breeds. Mahalanobis distances and procrustes distances values between the groups were obtained from CVA. A *p*-value < 0.05 was considered statistically significant.

## 3. Results

As a result of PCA analysis for dorsal view, 24 PCs were found. Here, PC1 explained the highest shape variation in relation to breeds 50.67%); PC2 accounted for 9.82% of shape variation, while PC3 represented 8.77% of shape variation (Table 2).

**Figure 2:**
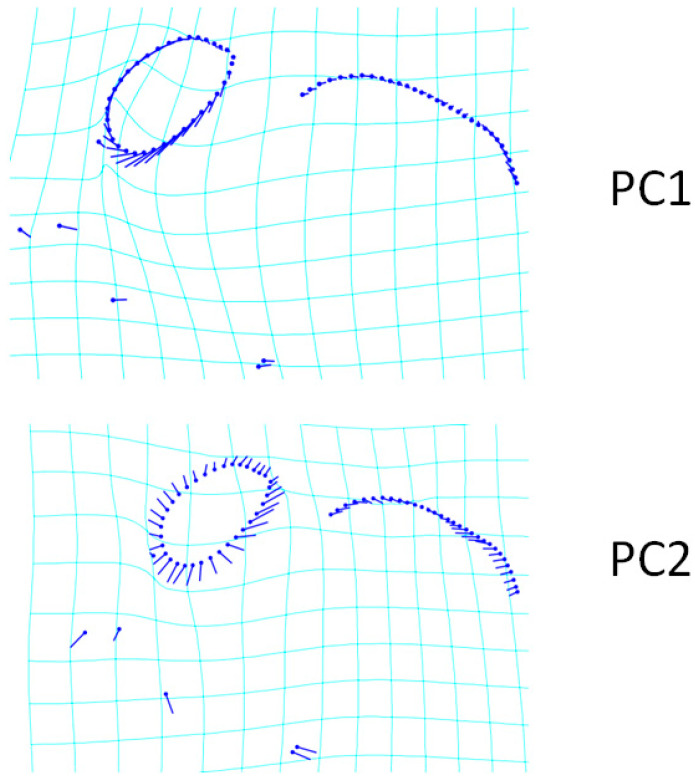
Transformation grid of changes in skull shape of PC1 (50.67%) and PC2 (9.82%) (dorsal view). Transformation grids illustrate the shape changes from the overall mean along PC1 and PC2. The length of the lines extending from the points represents the amount and direction of change.

The transformation grid of changes in the skull shape of PC1 and PC2 for the dorsal view is given in Figure 2. An increase in PC1 value represents a flatter head. As seen in Figure 2, the increase in PC1 indicates that the rostralmost point of the incisive bone and nasal bone are situated more caudally. It also showed that the cranio-medial edge of the orbit was more backward with increasing PC1 value. This represented a narrower orbital boundary. The change in the shape of the temporal fossa was relatively insignificant. The most distinct shape change in PC2 was at the orbital border. The increase in PC2 value represented a wider orbital pit in the dorsal view. In addition, as the PC2 value increased and the orbital boundary expanded, the nuchal crest approached the orbit. In other words, in the skull of Scottish Folds with a high PC2 value, the orbit was closer to the caudal border of the skull than in other breeds.

**Figure 3:**
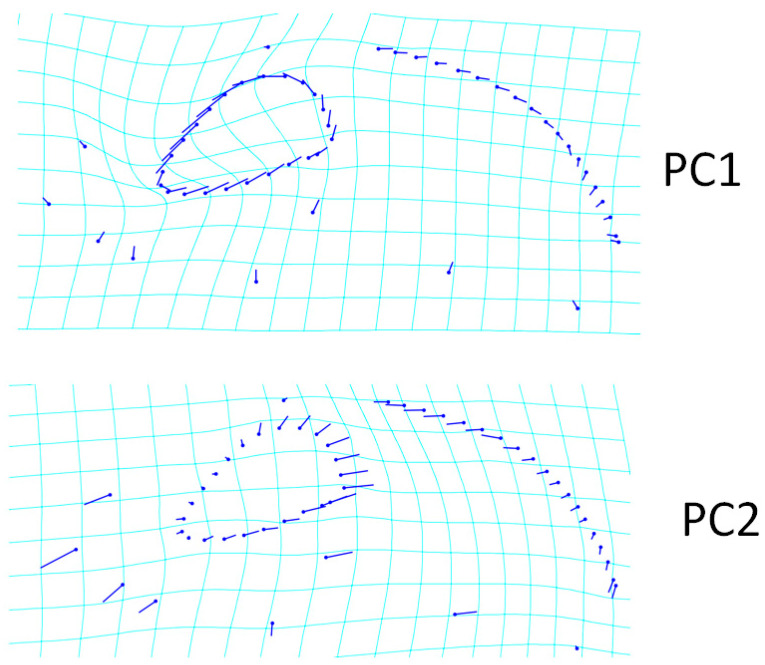
Transformation grid of changes in skull shape of PC1 (32.39%) and PC2 (18.45%) (lateral view). Transformation grids illustrate the shape changes from the overall mean along PC1 and PC2. The length of the lines extending from the points represents the amount and direction of change.

A total of 11 landmarks and 41 semilandmarks were used for the lateral view (Figure 3). As a result of PCA analysis, 24 PCs were found. The PC1 value for the lateral view was 32.39%, which explained the highest shape variation between breeds; PC2 accounted for 18.45% of shape variation, while PC3 accounted for 16.33% of shape variation (Table 2). The increase in PC1 value for the lateral view represented an upward change in the shape of landmarks. In addition, with increasing PC1 value, the caudal border of the orbit was further back. In the PC2 value, there was a forward change in landmarks. With increasing PC2 value, the facial bones (nasal and incisive) were closer to the orbit. In addition, the increased PC2 value represented the wider orbital boundary (Figure 3). Furthermore, an increase in PC2 value represented a narrower squamous part of the occipital bone.

**Figure 4:**
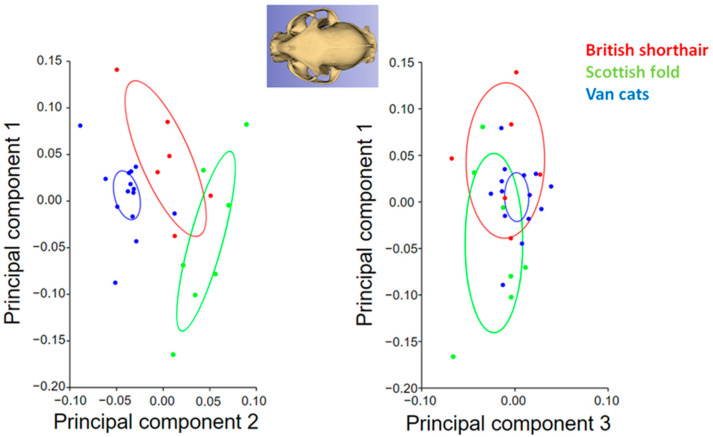
Principal component analysis scatter plot comparing skull morphology of cat breeds (dorsal view). Ellipses represent 95% confidence intervals around the means.

A principal component analysis scatter plot comparing the skull morphology of cat breeds for the dorsal view is given in Figure 4. The PC1 values of British Shorthairs were higher than other breeds. The PC1 value was low in Van cats, but the PC2 value was high in Van cats. In addition, the breed with the least variation in shape (for PC1, PC2, and PC3) was the Van cat. The average shape variation in the Van cat was smaller than the other breeds for the dorsal view. 

**Figure 5:**
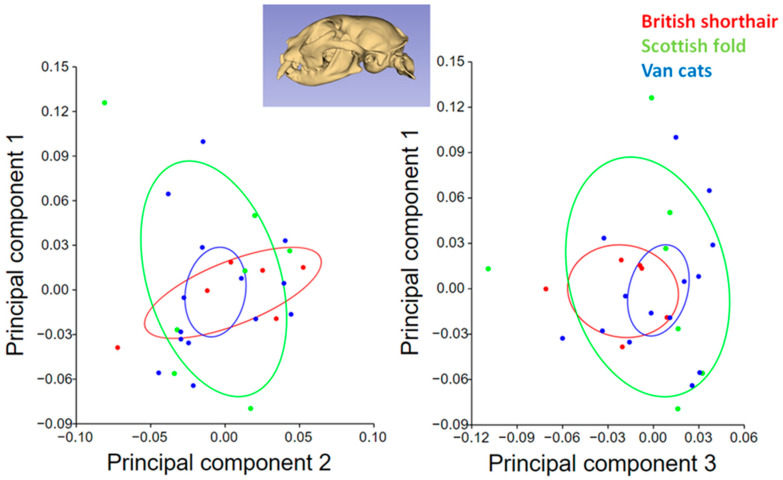
Principal component analysis scatter plot comparing skull morphology of cat breeds (lateral view). Ellipses represent 95% confidence intervals around the means.

A principal component analysis scatter plot comparing the skull morphology of cat breeds for the lateral view is given in Figure 5. Shape variations were greater in the lateral view than in the dorsal view. Shape variation was less in the British Shorthair than in other breeds for PC2. Shape variation explained by PC3 was less in Van cats than in other breeds. In the Scottish fold, the shape variability captured by PC1 and PC3 was greater than in other breeds.

Centroid size and shape differences were analyzed between cat breeds by procrustes ANOVA (Table 3). It was seen that the centroid size difference between the breeds was statistically insignificant. However, the differences in shape were statistically significant for both the dorsal and lateral views.

Mahalanobis distances and procrustes distances values and *p*-values are given in Table 4 (10,000 permutations). Mahalanobis distances between groups were statistically significant for both dorsal view and lateral view. However, procrustes distances were statistically significant only for the dorsal view. Procrustes distances for lateral view were statistically insignificant.

Abbreviations are as follows: MD, Mahalanobis distances among the group; MD-P, *p*-values from permutation tests (10,000 permutation rounds) for Mahalanobis distances among the group; PD, procrustes distances among the group; PD-P, *p*-values from permutation tests (10,000 permutation rounds) for procrustes distances among the group.

**Figure 6:**
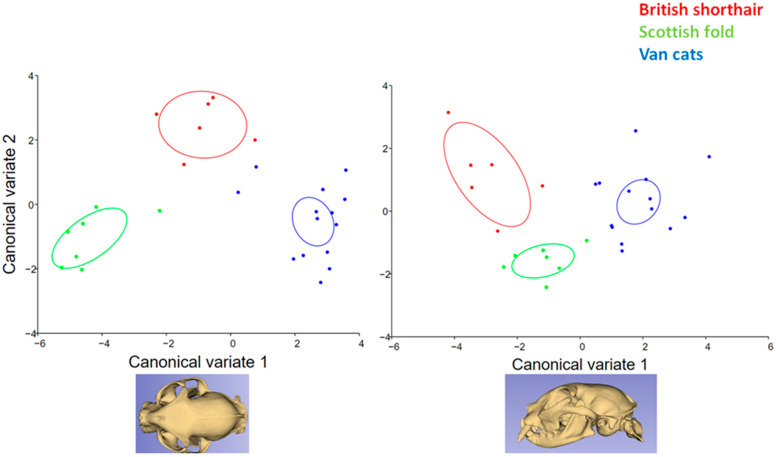
Frequency of distribution for CV1 and CV2 of cat skulls (n: 27) (*p* < 0.0001 from 10,000 permutation rounds for procrustes distances among groups).

In the canonical variate results, it was seen that the cat breeds were separated from each other (Figure 6). The Scottish Fold had low CV1 and CV2. The CV1 value of Van cats was higher than other breeds. The CV2 and CV3 values were higher in British Shorthairs.

**Figure 7:**
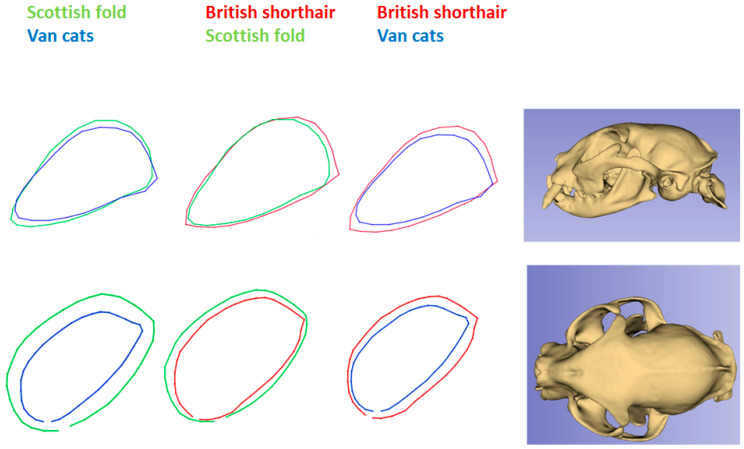
Wire-frame warp plots of changes in orbit shape of cat breeds for dorsal and lateral views.

Wire-frame warp plots of changes in the orbit shape of cat breeds for dorsal and lateral views are given in Figure 7. The British Shorthair had the widest orbital border in the lateral view. In the dorsal view, the Scottish Fold had the widest orbital border. The orbital border of Van cats was narrower in both dorsal and lateral views.

## 4. Discussion

The skulls of animals, including cats, undergo natural variability and evolutionary processes [20]. In domesticated animals, artificial selection is based on aesthetic factors and leads to the creation of diverse breeds [21]. Kruger et al. [22] showed that the cranial capacity of domestic cats is smaller than that of wildcats. The authors explain this fact with a more vaulted frontal portion of the skull and caudal displacement of the zygomatic process of the frontal bone. Breeding selection of companion animals is often guided by anthropocentric considerations [23]. A good example is the Scottish Fold, whose broadly-spaced eyes give the Scottish Fold a “sweet expression”.

In our research, it has been proven that the shape of the skull of each of the three cat breeds studied shows distinct characteristics. In the canonical variate analysis, it was observed that the breeds were separated from each other. However, there are some similarities between the British Shorthair and Scottish Fold, and Van cats occupy more distant areas in the charts. These results reflect the belonging of the examined cats to different morphotypes. The British Shorthair and Scottish Fold belong to brachycephalic cats, while the Van cat is a mesocephalic breed, with a morphology more similar to its wild ancestors.

Shape variability was lesser in the British Shorthair than in other breeds. This proves the morphological stabilization of the breed standard. The British Shorthair is possibly the oldest cat breed in Great Britain [7]. The Scottish Fold is a relatively young breed (bred around 1960). Due to inbreeding, it is allowed to cross with British Shorthair and British Longhair. As such, it is not surprising that the two breeds are so close together on the chart. Differences mainly concerned the orbit. In the skull of Scottish Folds, the orbit was located more caudally than in other breeds. The British Shorthair had the largest orbital ring in the lateral projection. In dorsal view, the orbit appeared largest in the Scottish Fold. The orbital ring of Van cats was the smallest in both dorsal and lateral views. This makes its skull similar to that of wild cats. The Fertile Crescent is credited with the domestication of the cat [24] and Lake Van lies on the outskirts of this land. The Turkish Van is a unique cat breed that was created naturally, without human intervention. As such, it can be considered a Turkish native breed [6].

The dorsal PC1 value explained more shape variation than the lateral PC1. For this projection, PC1 explained 50.67% of the total variation. For the lateral view, PC1 explained 32.39% of the total variation. Centroid size differences between breeds were statistically insignificant. However, the differences in shape were statistically significant for both the dorsal and lateral views.

There are studies in which shape analysis is applied to different parts of the cat skull. It has been proven that the process of domestication of the cat entails, among other things, the shortening of the neurocranium in its dorsal part [25]. A shorter skull also means a shorter external sagittal crest, which is the point of attachment of the temporal muscles. Domestication has radically changed the cat’s environmental conditions related to food acquisition. Hunting, although it remains one of the leading instincts, no longer determines survival. Huizing et al. [26] examined the morphological variations of the occipital bone in cats of 14 different breeds. They stated that Persian cats had a higher percentage of cerebellar crowds or hernias than all other breeds. However, they found no significant differences. Kunzel et al. [27] confirmed the phenotypically distinct skull formation in cats. Widely applicable today are breed standards that promote increased brachycephaly in cats, which has the potential to negatively impact their welfare, and potential buyers of brachycephalic cat breeds should be made aware of the risks of their conformation [28,29].

In our study, conducted on British Shorthair, Scottish Fold, and Van cats, geometric morphometric analysis of the skull was performed and important differences between these three breeds were found in the orbit. Geometric morphometrics is thought to effectively reveal the difference between animal species, breeds, and sexes. Christiansen [30] emphasized the morphological shape analysis of the skull in cats to formulate evolutionary hypotheses. Therefore, it is believed that the study of geometric morphometry can help answer the authors’ hypotheses not only in terms of anatomy but also in the development and evolution of living organisms.

Morphometric studies can reveal the size difference between samples. However, it is not sufficient to explain the variations that are not related to size [31]. Size differences can be seen in the competitive ecological pressures of animals [32]. Linear measurements can be used to reveal sexual dimorphism [33,34,35,36]. However, morphometric results may not give all the answers about shape. The size variation only affects the allometric variability of the cat’s skull and, therefore, cannot explain the entire range of morphological variability [31]. In geometric morphometrics, similarities and differences can be investigated in the morphologic patterns of the samples. For example, skull shape variations in wild cats living on different continents or cats with different hunting techniques can be examined and discussed. The cats used in this study represent similar environmental and lifestyle conditions. Although they have lived in the same geographical area for over 100 years and have very similar eating habits, they still have different variations in the shape of the skull. This could be revealed using geometric morphometric methods. Wąsowicz et al. [37] performed morphological and morphometric analysis of the occipital squama and the foramen magnum in a European cat. In the study, two categories were distinguished in the morphology of the occipital squama; the first was characterized by a form close to an isosceles triangle with the base directed to the bottom. However, in our study, there was no clear difference in shape between cat breeds in the squamous part of the occipital bone. An increase in PC2 value in the lateral view represented a narrower squamous part of the occipital bone.

Geometric morphometrics has been adopted to explain the evolutionary trends in the skull shape in monkeys [38]. It also allows us to understand the influence of diet on determining the shape of the skull in related carnivorous species. [39]. The shape of the orbit has also been reported to exhibit sexual dimorphism in humans [40]. Xiang et al. [41] studied the differences in the shapes of the orbits in different human populations. In the study presented here, an orbit shape analysis was performed to reveal the differences between cat breeds, and it proved to be effective.

## 5. Conclusions

The dorsal view was found to be more successful in breed discrimination. It was observed that the difference between breeds mainly concerned the orbit shape. Geometric morphometrics was found to be successful in distinguishing between cat breeds. The results of this research can be a reference for future studies concerning the skull of cats.

## Figures and Tables

**Table 1:** Cats which were used in the study.

Species	Female	Male	The Average Age (Years)	The Average Weight (kg)
British Shorthair	4	2	2.33	3.68
Scottish Fold	4	3	3.71	4.03
Van cats	7	7	4.5	5.61

**Table 2:** Five PCs that explain the highest variation for dorsal and lateral views.

PCA	Dorsal View		Lateral View	
	Eigenvalues	% Variance	Eigenvalues	% Variance
PC1	0.00345538	50.668	0.00245058	32.394
PC2	0.00066936	9.815	0.00139585	18.452
PC3	0.00059811	8.770	0.00123523	16.329
PC4	0.00047461	6.959	0.00057177	7.558
PC5	0.00042940	6.296	0.00030508	4.033

**Table 3:** Centroid size and standard deviations of cat skulls.

Individuals			F	*p*-Value
Breeds	Dorsal view	Centroid size	0.58	0.5679
		Shape	5.93	<0.0001
	Lateral view	Centroid size	0.20	0.8201
		Shape	1.34	0.0015

**Table 4:** Mahalanobis distances and procrustes distances values and *p*-values for the cat skull.

	MD	MD-P	PD	PD-P
Dorsal view	4.9804	<0.0001	0.0326	<0.0001
Lateral view	3.2764	<0.0001	0.0365	0.2066

## Data Availability

Not applicable.
